# Mini-open transosseous repair with bursal augmentation improves outcomes in massive rotator cuff tears

**DOI:** 10.1038/s41598-025-85520-2

**Published:** 2025-01-17

**Authors:** Yasser El Safoury, Ahmed O. Sabry

**Affiliations:** Department of Orthopedics and Traumatology, KasrAlAinyFacultyofMedicine, Cairo University, Al- Manial, Cairo, Egypt

**Keywords:** Mini-open, Massive rotator cuff tear, Biological bursa, Muscle, Tendons, Outcomes research

## Abstract

Treatment of Massive rotator cuff tears (MRCT) is difficult, with high rates of retears. Using biological augmentation in the form of the highly vascular subacromial bursa, was used to improve tendon healing. This work aimed to evaluate the results of arthroscopic guided mini-open transosseous repair with bursal augmentation in the treatment of MRCTs in a five-step approach. Forty-eight patients, with a mean age of 63.15 years, were treated with this technique. The patients were evaluated with the constant, UCLA and VAS scores. Plain X-rays were performed to evaluate the CSA and MRI was done to confirm the diagnosis of MRCT and determine the degree of fatty degeneration. Ultrasound was done at 1 year post-operative to determine any retears. The mean follow-up period was 29 months ± 4.95. The Constant and UCLA mean scores improved from (52.52) to (89) and (13.2) to (30.5) respectively (*p* < 0.0001). The post-operative active flexion and abduction improved from a mean of (112° to 170°) and (136.2° to 167°) respectively, while ER improved from (62.8° to 70°) with their p values (*p* < 0.0001). Pain improved from a mean VAS of (5.85) to (0.5) (*p* < 0.0001). No deterioration of function was noted throughout the follow-up period, and no retears occurred on post-operative ultrasound evaluation. Mini-open transosseous repair with bursal augmentation in the treatment of MRCT is an effective and low-cost method that achieves satisfactory results with no retears.

## Introduction

Massive rotator cuff tears (MRCT) present significant treatment challenges, often leading to a high incidence of recurrent tears post-surgery^1–3^. The definition of MRCT varies^4–6^, but Gerber’s classification, which considers the number of tendons involved, is most commonly referenced in the literature^7–9^. It has been reported that the retear rate following surgical treatment ranges from 17.6 to 94% for large and massive tears^2,10–13^. Most of these retears occur in the first two years of follow-up, as mentioned in a recent meta-analysis study^14^. This high failure rate persists despite the introduction of arthroscopic techniques, with studies showing no statistically significant difference in outcomes compared to mini-open repairs^15–17^. Massive tears may also show varying grades of fatty degeneration which may improve after successful repair indicating that fatty degeneration is not an absolute contraindication for repair^18,19^. Massive tears compromise the biological healing environment, which may be a major cause of re-rupture. Several methods have been proposed to augment biological healing in MRCT repairs, including Subacromial balloon spacers^20^, Synthetic and bioengineered grafts^21^, tendon allograft patch augmentation^22^, Dermal allografts^23^, Applying platelet-rich plasma (PRP) over the repair site^24^, Biceps Tendon Autograft^25^ and the usage of Subacromial Bursa^26–29^. The subacromial bursa proved to possess considerable tendon-healing potential which may be owed to its pluripotent stem cells^30^, extensive blood supply^31^, which may enhance healing, and its lubricating property, reducing friction over the upper surface of the rotator cuff^32^. Other benefits include the ease of use and almost no additional expenses.

However, standardized surgical techniques for bursal mobilization, harvesting, and vascularity preservation remain lacking in the literature. ^.^Preoperative imaging can predict the reparability of MRCTs^33–35^, yet intraoperative assessment of tendon quality and mobility often serves as the definitive determinant^1,36–38^. There is ongoing debate regarding the necessity of acromioplasty and tenotomy of the long head of the biceps tendon (LHBT) in treating MRCTs, with varying practices reported^39–42^. Transosseous (TO) repair originally described by McLaughlin in 1944^43^ has been used in arthroscopic and mini-open rotator cuff repair by many authors and proved to be a better option for repair due to their lower cost, better healing interface, and avoidance of the complications regarding anchor failure^44^. Because most clinical studies in the literature do not present the surgical techniques for MRCT repairs in a step-by-step approach it is difficult for researchers to compare the results. This study aims to evaluate the clinical and radiological outcomes of a mini-open transosseous repair with bursal augmentation in treating MRCTs, and to establish a standardized five-step surgical approach for this technique.

## Material

Forty-eight patients with massive rotator cuff tears were treated with our standardized five-step technique of arthroscopic guided mini-open transosseous repair with bursal augmentation. MRCT was defined as involving two or more tendons according to Gerber classification^5^. Exclusion criteria were designed to ensure a homogeneous sample and remove any associations that may affect function after treatment. They included patients with revision cases, rotator cuff tears associated with neurological lesions, osteoarthritis of the glenohumeral joint classified as grades 2 or 3 by the Samilson-Prieto classification^45^, rheumatoid arthritis, or postinfectious conditions. These patients were referred to our university hospital after the failure of several months of conservative treatment and had undergone surgery between September 2019 and December 2023.

### Ethical consideration

All patients provided informed consent, and the study was conducted in accordance with the Declaration of Helsinki. The experimental protocols were approved by the institutional review board of Cairo University Hospital. The study was also registered on ClinicalTrials.gov with the identifier: NCT06242158.

**Fig. 1:**
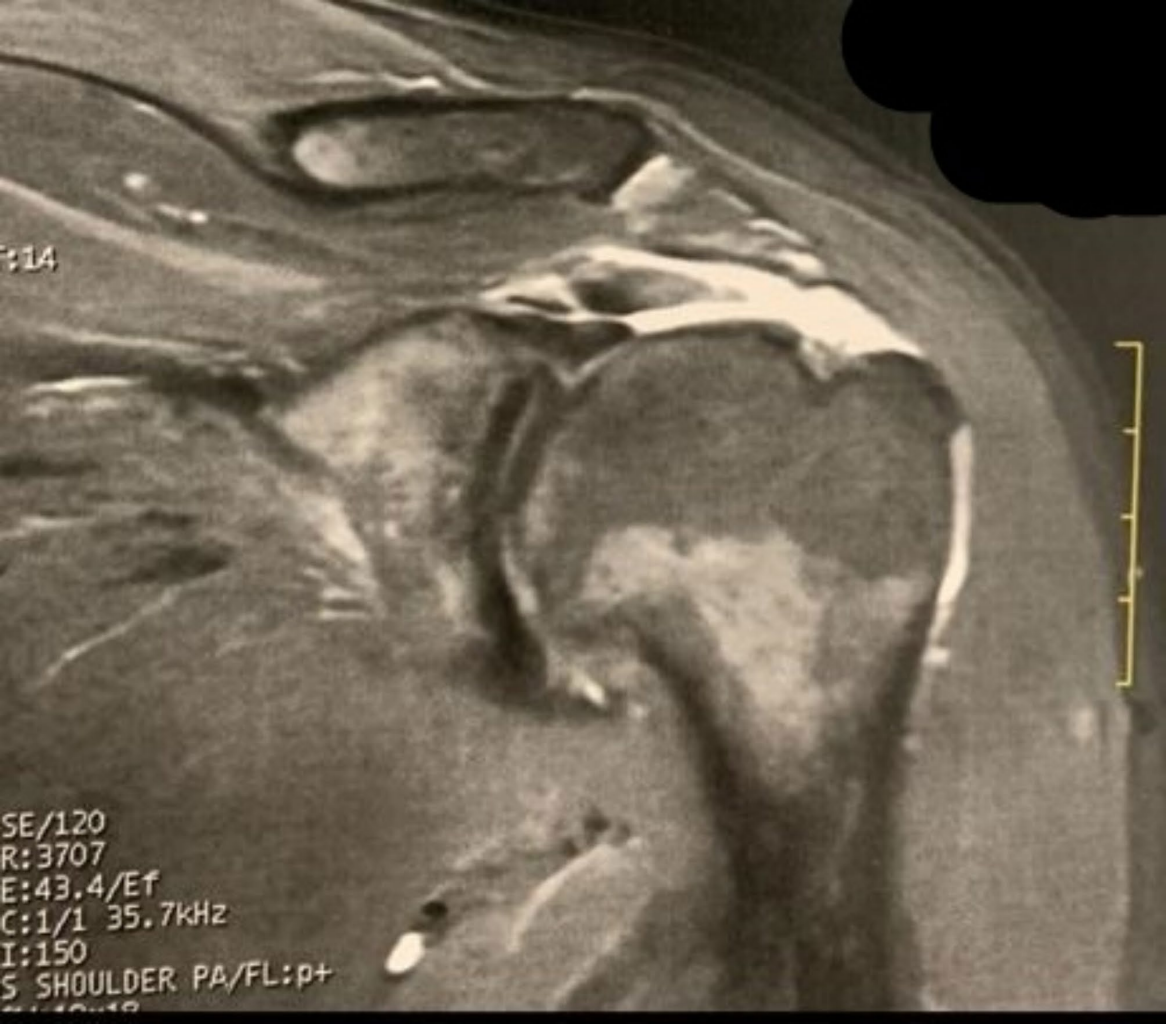
MRI coronal cut section showing a complete tear of the suprapinatus tendon.

The study group included (22 men and 26 women) with a mean age of 63.15 years with a range of (53–69) years**.** Twelve of the patients were diabetics and one was diagnosed with chronic kidney disease that required regular dialysis. Patients were evaluated clinically for the active range of movements of the shoulder in foreword flexion, abduction, and internal and external rotation. The Constant score and University of California-Los Angeles (UCLA) scores were used for functional evaluation. The Constant Score assessed overall shoulder function, incorporating pain, range of motion, strength, and daily activity levels. The UCLA Shoulder Score evaluated pain, function, active range of motion, and patient satisfaction. The Visual Analogue Scale (VAS) score was used to assess pain. An outpatient ultrasound was used to evaluate the condition of the rotator cuff. Standard plain X-rays of the shoulder were performed to assess the Critical shoulder angle (CSA)^46^. Nineteen (39%) patients had associated type II or III Bigliani acromions. MRI was performed to confirm diagnoses of MRCT (see Fig. 1) and to evaluate the degree of fatty degeneration according to the Goutallier classification^47^.

Most of our cases (73%) presented with Collins-type D tears. Regarding fatty degeneration, 36 patients (75%) exhibited grade 1, 2, or 3 fatty degeneration, while only 12 patients (25%) showed no signs of fatty degeneration.

The demographic data are summarized in **(**Table 1**)**

**Table 1:** Shows the baseline data of the included patients.

Number of cases	Age (years)	Gender		Side		Dominance		Follow-up period (months)
N = 48	63.15 ± 4.38 (53–69)	Men	22	Right	22	Dominant	21	29.46 ± 4.95 (24–38)
		Women	26	Left	26	Non-dominant	27	

Type of tear (Collins classification)		Fatty degeneration (Goutallier)		Bigliani Acromions		Tenotomy or Tenodesis		
A	6 (12.5%)	0	12 (25%)	Type I	29 (60.42%)	None	38	
B	6 (12.5%)	1	19 (39.58%)					
C	1 (2.08%)	2	11 (22.92%)	Type II	8 (16.67%)	Tenotomy	8	
D	35 (72.92%)	3	6 (12.5%)					
E	0	4	0	Type III	11 (22.92%)	Tenodesis	2	

### Surgical technique

In conjunction with general anesthesia, an interscalene brachial plexus block was given for the operation. Patients were placed in a semisetting position. Conventional posterior, lateral, and anterolateral portals for arthroscopy were used for both glenohumeral and subacromial spaces. The posterior portal was made 2 cm inferior and medial to the posterolateral corner of the acromion. In the same anteroposterior plane as the distal clavicle, the anterolateral portal is situated 2 cm lateral to the distal acromion. Off the anterolateral margin of the acromion, an accessory lateral portal was formed to facilitate instrumentation if necessary.

Surgery was performed in a five-step approach including:

#### Step one: Identifying the type of tear, if repairable and reducible

A posterior subacromial portal was used to identify the size and quality of the rotator cuff tear. Using a shaver, minimal removal of the bursa and gentle mobilization of the tendon was done. A grasper was then applied to pull the tendon to determine if it was reducible to the footprint area. If the tendon was friable or if the released pulled tendon did not cover more than 50% of the bare bone of the footprint, a shift to tendon transfer surgery would be decided.

#### Step two: Preparing both the subacromial space and footprint together with identifying the biceps tendon pathology

Acromioplasty, with removing the anterior and lateral part of the acromion was performed in all type II and III Bigliani acromions, to reach a capacious subacromial space in different positions testing shoulder forward flexion and abduction. Minimal removal of the coracoacromial ligament was considered to prevent superior instability together with minimal removal of the anterior deltoid fibers to prevent further detachment when later performing an anterolateral mini-open approach. The LHBT was palpated and mobilized, looking for any signs of degeneration and fraying or instability. The LHBT was inspected either through the tear using the same subacromial portal or through the glenohumeral portal. For patients aged 65 years or older, when indicated, tenotomy was performed close to the origin of the glenoid labrum. For active and younger patients, tenodesis was added using non-absorbable sutures to fix the LHBT to the insertion of the pectoralis major tendon by a separate incision. Lastly, the footprint was prepared with a slow-speed burr.

#### Step three: Mini open anterolateral surgical approach

**Fig. 2:**
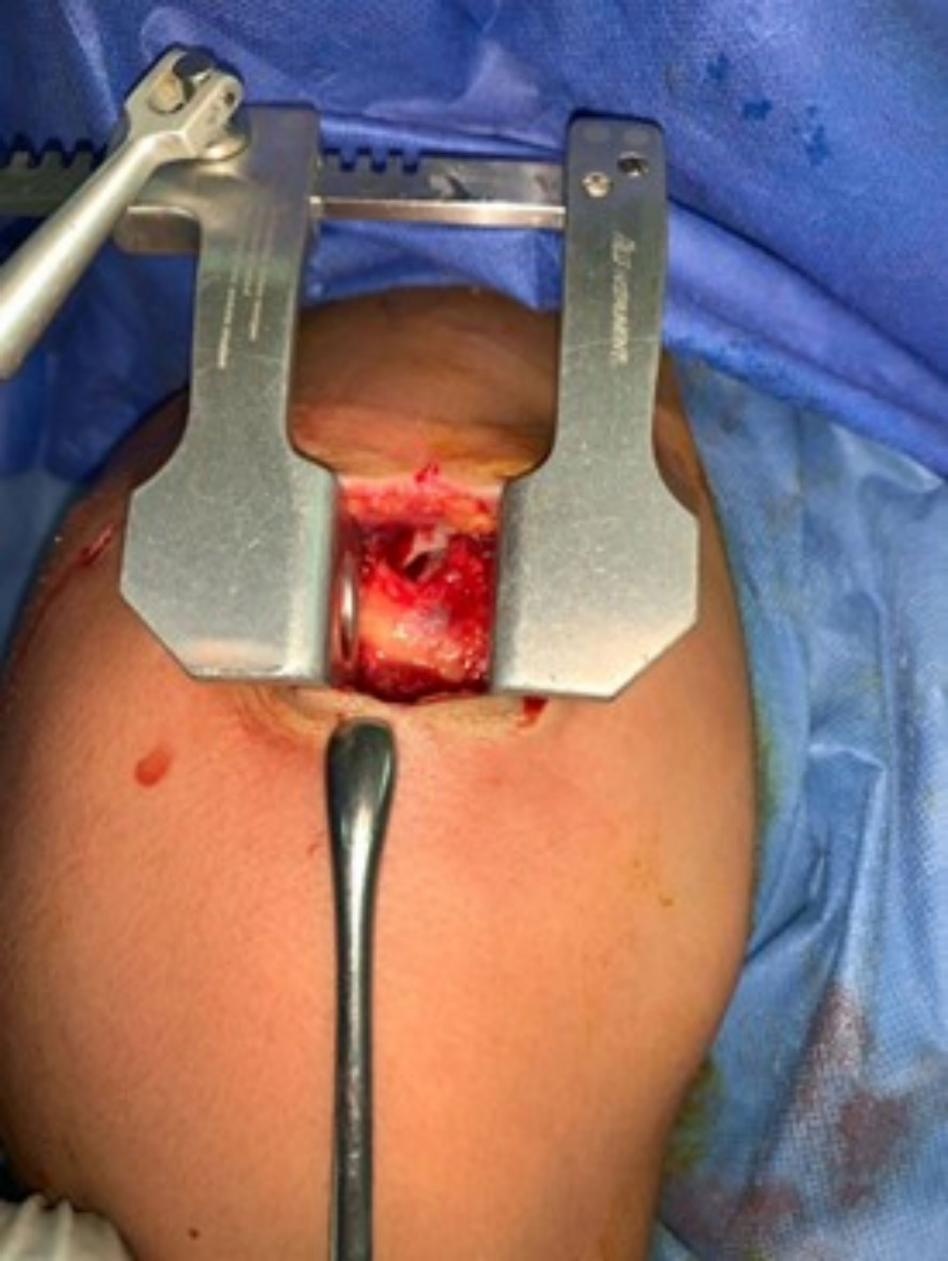
Photograph showing the exposure of the torn tendon after applying a blunt retractor to retract the deltoid muscle. A Cobb dissector is a useful tool to gently mobilize the tendon to its footprint.

An anterolateral mini-open approach was used through a longitudinal 4 cm skin incision aligned with the anterior acromion. Followed by splitting the deltoid muscle between the anterior and middle fibers through the anterolateral raphe. A blunt deltoid retractor was applied, and the involvement and configuration of the torn tendon were confirmed and visualized by rotating the arm in different positions. (see Fig. 2).

#### Step four: Transosseous repair of rotator cuff

**Fig. 3:**
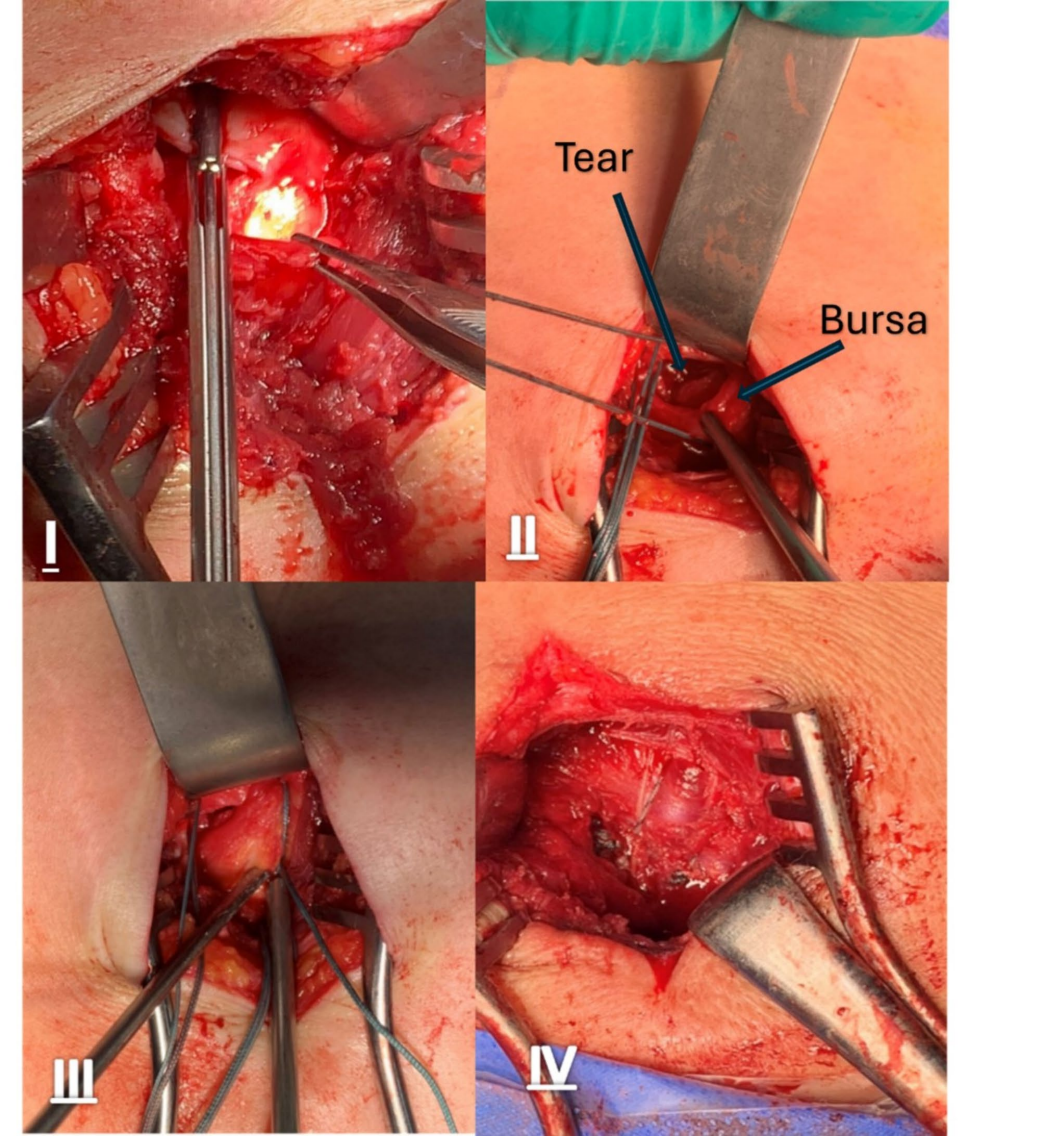
I) Photo shows the rotator cuff tear and the subacromial bursa after mobilization; II) Passing the sutures through the tendon and bone then retrieving its end superficial to the bursa; III) Pushing the sliding knot at the lateral side of the humerus so the bursa overlaps the repair; IV) Final coverage of the tendon with the bursa.

To ensure even tension distribution, three or four size two FiberWire® sutures were first passed equidistant through the tendon with horizontal stitches used when necessary. The giant needle was then introduced taking one strand of the suture to penetrate the bone at equidistance on the footprint and emerging from the skin, about 5 cm from the tip of the greater tuberosity. Having the shoulder free allows movements of the humerus to help localize the whole bare area according to the anatomical footprints as well as showing any anterosuperior or posterosuperior tears. The sutures passed using the Giant needle were retrieved subdeltoid, superficial to the bursa by a right-angle hook. Before tightening the sutures, the subacromial bursa was mobilized gently from the medial and posterior sides toward the tendon ends. (see Fig. 3-I to IV).

#### Step five: Biological bursa augmentation

**Fig. 4:**
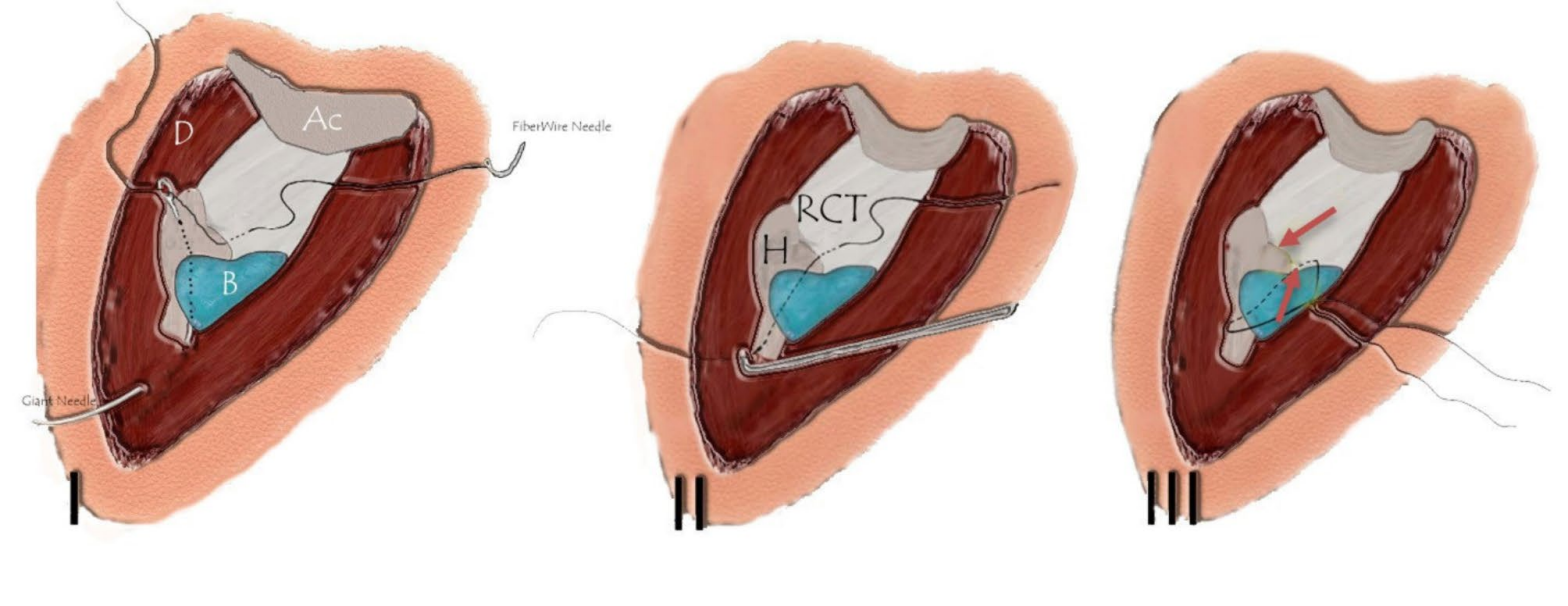
I) Diagram shows passing the FiberWire® through the tendon and then a giant needle is introduced to penetrate the bone with the distal strand of the sutures; II) The suture is pulled subdeltoid superficial to the bursa with a right-angled hook; III) Tightening the knot pushes the bursa to cover the repair without extra sutures. [Ac:Acromion; D:Deltoid; B:Bursa; RCT:Rotator Cuff Tendon; H:Humerus].

The subacromial bursa was used for biological augmentation of the repair by covering the repaired tendon. Mobilizing the subacromial bursa without jeopardizing its vascular supply is a critical step. The Cobb dissector proved to be a valuable tool, enabling blunt mobilization of the bursa while preserving the supplying vessels. This technique allows the bursa to be advanced from the medial and posterior sides to cover the localized repair site without causing any soft tissue trauma. This was done by pushing the sliding knot at the lateral side of the humerus so the bursa overlaps the repair. The sutures pull the tendon down to the bone and overlap the bursa without additional sutures. (see Fig. 4-I to III)

Postoperative care routine: Patients were fitted with abduction braces to ensure proper shoulder positioning and immobilization. Starting from the first week postoperatively, patients began passive range-of-motion (ROM) exercises and pendulum exercises to promote joint mobility and reduce stiffness.

At six weeks post-surgery, active-assisted and active free ROM exercises according to the pain tolerance of the patient were allowed. This phase aimed to gradually restore functional movement while avoiding undue stress on the healing tissues.

At three months postoperatively, patients started muscle-strengthening exercises and occupation therapy to return to their original jobs and sports.

## Results

The mean follow-up period for the patients was 29 months ± 4.95 with a range between 24 and 38 months. The functional scores, outpatient shoulder ultrasound, and VAS scores were recorded at six, twelve, and twenty-four months post-operatively (see Table 2).

**Table 2:** Shows the outcome data of the included patients.

	(VAS) score	Constant score	(UCLA) score	Active Anterior flexion angle degrees	Active abduction degrees	Active external rotation degrees	Active internal rotation degrees
Pre-operative	5.85 ± 1.3	52.52 ± 3.7	13.2 ± 3.7	112 ± 30	136.2 ± 20.4	62.8 ± 11	56.25 ± 5.78
Post-operative 6 months	2.18 ± 1.1	84.3 ± 3.3	26.8 ± 5.8	155 ± 28	160 ± 18	65 ± 6	59.27 ± 7.14
Post-operative 12 months	1 ± 0.66	87.6 ± 3.32	29.1 ± 4.4	168 ± 11	167 ± 14	69 ± 2	71.08 ± 6.4
Post-operative 24 months	0.5 ± 0.5	89 ± 3	30.5 ± 5.2	170 ± 9	167 ± 14	70 ± 2	71.1 ± 3.1

Results showed a mean improvement in Constant score from 52.52 ± 3.7 preoperatively to 89 ± 3 postoperatively, and was statistically significant (*p* < 0.001), with a 95% confidence interval (CI) for the difference ranging from 34.2 to 38.1. Similarly, the UCLA score improved from 13.2 ± 3.7 to 30.5 ± 5.2 (*p* < 0.001; 95% CI 16.1–18.3), and the VAS score decreased from 5.85 ± 1.3 to 0.5 ± 0.5 (*p* < 0.001; 95% CI −5.4 to −4.8).

For range of motion outcomes, active forward flexion improved from 112° ± 30 to 170° ± 9 (*p* < 0.001; 95% CI 50.3° to 63.8°), abduction from 136.2° ± 20.4 to 167° ± 14 (*p* < 0.001; 95% CI 26.5° to 37.3°), and external rotation from 62.8° ± 11 to 70° ± 2 (*p* < 0.001; 95% CI 5.8° to 9.6°). These statistical analyses indicate the significant functional improvements achieved with our technique.

All patients had a good level of internal rotation and could turn the dorsum of the hand to T12 or between their shoulder blades. Patients were able to return to their normal life and activities within an average of 6 months (See Table 2).

All patients showed complete healing with no deterioration of function or retears confirmed by ultrasound evaluation. None of our cases experienced complications such as elbow flexion weakness, deltoid function impairment, or axillary nerve injury after the repair of these massive rotator cuff tears.

## Discussion

MRCTs are challenging to treat due to the poor tissue quality, limited healing potential and the high retear rate^48^. Several surgical techniques have been proposed to address these issues however none of these techniques have shown consistent and satisfactory results, and each has its limitations and complications^37^. This work presents a novel technique that combines several advantages of different methods while avoiding their drawbacks. This technique of mini-open transosseous repair with bursal augmentation is presented in a standardized five-step approach to be easily reproducible and compared with other studies.

First, we performed arthroscopic identification of the tear and its reparability, which allowed for a precise assessment of the tear size, shape, location, and tissue quality. We did not consider preoperative limited flexion less than 90 degrees as an indication of tendon transfer as recommended by some authors^49,50^, and agreed with other authors that good results were achieved with successful repair and healing^51,52^. We did not consider fatty degeneration as an absolute indication for tendon transfer or reversed shoulder arthroplasty. We followed the recommendations of different studies that analyzed the relation between presurgical fatty degeneration of the rotator cuff and retears and showed no statistically significant difference^51,53^*.* In our work, the indication for shifting to other salvage procedures was arthroscopic identification of a non-repairable or non-reducible tendon to the footprint. The LHBT was also arthroscopically identified and was sacrificed only in cases with subluxation or dislocation, partial lesions affecting more than 25% of the thickness, or superior labrum lesions of types 2, 3, or 4^41,42^. Intraarticular tenotomy was performed for older patients with limited activities while tenodesis was performed for younger and more active patients. In this study, a total of 8 tenotomies and 2 tenodesis procedures were performed during the repair operation.

Second, we performed acromioplasty only in cases of Bigliani type II and III acromions, with a critical shoulder angle CSA of more than 35 degrees to decrease the re-rupture rates following the current consensus in the literature^39,54^. A total of 19 (39%) cases either had Bigliani type II or III acromions and underwent acromioplasty during repair. We believe that acromioplasty should be performed selectively and judiciously based on the acromion morphology and the tear characteristics. Special concerns were directed to remove as little as possible of either the coracoacromial ligament or anterior fibers of the Deltoid during acromioplasty to prevent later superior migration of the humeral head or deltoid fibers disruption.

Third, we used an anterolateral longitudinal mini-open approach to access the rotator cuff and perform the repair. This approach proved to be more favorable than the lateral approach as it allowed the identification of all types of tears with anterior or posterior extension^55^. Also, it preserves the deltoid muscle integrity and reduces the risk of deltoid detachment, by splitting the fibers at the raphe between the anterior and middle fibers.

Fourth, we performed the transosseous repair with a giant needle as described by Fleega^56^. Because of the large size of the core of the giant needle which may cause injury to the tendon or if there is a need to pass horizontal sutures in atrophied sections, we first passed the needle of the FiberWire® through the tendon side then used the giant needle to penetrate the bone under vision in its appropriate footprint site. Great care was taken not to pinch the long head of the biceps if it was preserved. Also, a meticulous suturing technique ensured smooth and even edges without any wrinkling. We believe that proper pull and direction of the tendon to cover the whole footprint is important for good functional results.

Fifth, we performed biological augmentation of the repair with the subacromial bursa, which is a novel and innovative technique that has not been reported before in MRCTs by a mini-open technique. The exact cause of retear was not fully discussed in the literature; however, trying to augment the repair with biological materials has improved results^21–24,26,57^. The rationale behind using the subacromial bursa to enhance the healing potential of the rotator cuff can be categorized into three primary functions. First, it serves as a source of mesenchymal stem cells thus creating a favorable environment for tissue regeneration and repair^58–61^. Second, its vascularity plays a crucial role in the healing process^31,62^, further supporting the repair process. Third, the bursa’s natural lubricating effect prevents adhesions and minimizes impingement on the acromial surface^29,32^. By covering the repaired cuff, the bursa reduces friction and shear forces under the acromion, thereby lowering the risk of adhesions and subsequent retears. Additionally, the use of the subacromial bursa as an autologous tissue is cost-effective, eliminating the need for additional graft materials and avoiding the risks associated with allografts or synthetic implants, such as immune reactions or implant failures. Although the use of the subacromial bursa proved to be effective, to our knowledge, the technique of dissection and mobilization either arthroscopic or mini-open was not thoroughly investigated. In this work, we described how to mobilize the bursa from medial and posterior with its blood supply to cover the repaired tendon with minimal sutures.

Patients were followed up for a mean of 29 months and evaluated as regards their clinical outcomes using the Constant-Murley score, the UCLA score, and the visual analog scale for pain and radiologically by shoulder ultrasound. This technique of mini-open transosseous repair with bursal augmentation resulted in significant improvements in pain, function, and range of motion, as well as no retear rates. These results are superior to those reported by previous studies using different techniques for massive RCTs in regards to the rates of retears of functional deterioration during follow-up^13,51,63–65^. Studies using arthroscopic techniques report retear rates ranging from 17.6 to 94% due to limited healing potential and anchor-related complications^2,10–13,66,67^. This transosseous repair avoids anchors entirely, eliminating anchor failure as a potential complication, while biological augmentation with the subacromial bursa proved to enhance healing, contributing to the absence of retears in our study. In this study, it was also observed that there was no deterioration of function reaching the final follow-up period. Regarding functional outcomes, our UCLA score improved from 13.2 to 30.5, demonstrating results comparable to those achieved with other techniques for MRCTs, as reported by several authors in the literature^11,68–70^. Our simpler, cost-effective mini-open approach offers similar outcomes, making it more accessible in resource-limited settings. Incorporating the bursa as biological augmentation in the repair of MRCT holds great potential. The preliminary evidence supports its effectiveness in enhancing tendon healing and improving clinical outcomes. Continued research and clinical trials will be crucial in establishing its role as a standard adjunct in rotator cuff repair surgeries. In our study, all procedures were performed by the same surgical team, led by the senior author, a highly experienced orthopedic surgeon specializing in the treatment of massive rotator cuff tears. This consistency in surgical expertise ensured procedural standardization and minimized variability in outcomes. This study divided the technique into a 5-step technique to be straightforward and reproducible.

This study has some limitations in the form of the lack of a control group. While our results demonstrate significant improvements in functional outcomes and the absence of retears with the mini-open transosseous repair and bursal augmentation technique, a direct comparison with alternative methods would provide stronger evidence for its efficacy. A control group using standard repair techniques, with or without other forms of biological augmentation, could highlight the specific contributions of bursal augmentation to healing and retear prevention. Therefore, further studies with larger cohorts, randomized controlled designs, and longer follow-up periods are needed to confirm the efficacy and safety of this technique. Future research could also explore the incorporation of nanomaterials into the subacromial bursa during augmentation to potentially enhance its regenerative properties^71^. Diagnosing MRCTs accurately is challenging, particularly regarding classification and factors like tear patterns, tendon quality, and fatty degeneration. Implementing artificial intelligence in imaging could improve diagnostic precision. Finally, postoperative evaluations relied on ultrasound, which, while practical, may lack the sensitivity of MRI for detecting subtle structural changes.

In conclusion, this technique of mini-open transosseous repair with bursal augmentation in the treatment of MRCT is a feasible, safe, and effective method that achieves satisfactory functional outcomes with no retears.

## Data Availability

The data supporting the findings of this study are available upon reasonable request from the corresponding author.
